# Patient expectations of benefit from systemic treatments for metastatic prostate cancer

**DOI:** 10.1002/cam4.2783

**Published:** 2019-12-16

**Authors:** Laura B. Oswald, Rachel Kasimer, Katherine Rappazzo, Angela J. Fought, David F. Penson, Alicia K. Morgans

**Affiliations:** ^1^ Northwestern University Feinberg School of Medicine Chicago IL USA; ^2^ Johns Hopkins University Baltimore MD USA; ^3^ University of Colorado Denver CO USA; ^4^ Vanderbilt University Medical Center Nashville TN USA

**Keywords:** metastatic disease, prognostic understanding, prostate cancer, systemic treatments, treatment expectations

## Abstract

**Background:**

Metastatic prostate cancer is incurable, but systemic therapies can improve quality of life and prolong survival. Accurate perceptions of treatment risks and benefits are vital as patients with metastatic disease make treatment decisions. We assessed treatment‐related expectations for benefit among patients with metastatic prostate cancer and explored associated sociodemographic characteristics.

**Methods:**

Men with metastatic prostate cancer (N = 100) completed surveys assessing their treatment‐related expectations for cancer cure, symptom relief, and prolonged life expectancy. Frequencies were used to describe the proportions of reported expectations. Fisher's exact tests were used to assess the associations of sociodemographic characteristics with treatment expectations.

**Results:**

One third (33%) of participants believed treatment was at least a little likely to cure their metastatic cancer. Most participants believed treatment could provide symptom relief (76%) and extend life expectancy (95%). Among participants reporting that cancer cure was at least a little likely vs not at all, more men identified as non‐white (24% vs 5%; *P *= .01), self‐reported good health (90% vs 58%; *P *< .01), and had greater optimism (78% vs 47%; *P *< .01). Among participants reporting that symptom relief was at least a little likely vs not at all, more men were less than 70 years old (62% vs 0%; *P *= .01).

**Conclusion:**

A large proportion of patients with metastatic prostate cancer reported beliefs inconsistent with understanding that treatment was not curative. Race, better self‐reported health, and greater optimism were related to unrealistic expectations. Efforts to ensure alignment of patient and clinician expectations may facilitate more effective shared decision‐making for treating metastatic disease.

## Graphical abstract

Despite having incurable disease, approximately one‐third of men with metastatic prostate cancer hold the inaccurate belief that systemic treatment is at least a little likely to cure their cancer. Being of non‐white race, better self‐perceived health, and greater optimism were associated with this inaccurate belief.

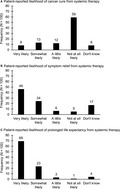

## 1 INTRODUCTION

Prostate cancer is the most common cancer among men in the United States, accounting for 20% of new cancer diagnoses annually.^1^ Early detection and advances in cancer treatments have led to improved 5‐year relative survival for men with early‐stage disease so that it now approaches 100%. However, for men with metastatic prostate cancer, 5‐year relative survival is 30%, and metastatic prostate cancer remains incurable.^1^ Systemic therapies (eg, chemotherapy, hormonal therapy, immunotherapy, and radiopharmaceuticals) can be used to reduce disease‐related symptom burden and prolong life among patients with metastatic prostate cancer,^2^, ^3^ and indeed survival outcomes have improved for men with metastatic prostate cancer in recent years.^4^ However, systemic treatments are associated with significant toxicities and reduced quality of life for some patients.^5^ Understanding the potential risks and benefits of therapies to treat metastatic prostate cancer is critical as men make decisions about their care, particularly in the context of making decisions for palliative treatment for incurable disease.

Patients with metastatic cancer often report expected benefits from systemic therapies that are not aligned with physician expectations. For example, in a large prospective cohort study of nearly 1200 patients undergoing chemotherapy for incurable cancer, 81% of patients with colorectal cancer and 69% of patients with lung cancer endorsed beliefs inconsistent with the understanding that treatment was not at all likely to cure their cancer.^6^ Other studies have found that 50% of patients with incurable gastrointestinal cancers believed that the goal of initiating chemotherapy was to cure their disease,^7^ and 32% of patients with incurable nonsmall‐cell lung cancer believed their cancer was curable.^8^ A recent study demonstrated that almost a quarter of patients with metastatic genitourinary cancers who were initiating immunotherapy (including men with metastatic prostate cancer) reported inaccurate expectations for potential cure, and inaccurate expectations were related to higher anxiety.^9^

In addition, multiple studies report that patients with incurable cancer overestimate their life expectancy.^10^, ^11^, ^12^ Recently, a study of approximately 300 patients with incurable cancer who were willing to estimate their life expectancy explored factors associated with patient‐reported life expectancy.^12^ When patients did not recall a discussion with their treating physicians regarding prognosis, patients reported life expectancies a median of 3 years longer than their peers who did recall prognostic discussions (median 4 years life expectancy vs one year, respectively). Longer patient‐reported life expectancy was in turn related to less likelihood of do‐not‐resuscitate orders and greater preference for life‐prolonging vs comfort‐oriented care.^12^ Thus, misconceptions related to the potential benefits of systemic therapies and life expectancy contribute to poorly informed decision‐making among patients with incurable cancer and may lead patients to accept toxic therapies that are not consistent with their overall goals of care or personal preferences.

Understanding the expectations that men with metastatic prostate cancer have regarding potential benefits of systemic therapies is increasingly important, as men with metastatic disease often receive multiple lines of treatment and live with the consequences of their treatment decisions for longer than ever before.^4^ Yet, the expectations for cure and other benefits of systemic therapies have not been described among men with metastatic prostate cancer. We sought to address this gap in the literature by characterizing the expectations for cure and palliation from systemic therapies among men with incurable metastatic prostate cancer. In addition, we sought to identify sociodemographic patient characteristics associated with these beliefs.

## 2 METHODS

### 2.1 Participants and procedures

The Institutional Review Board (IRB) at Vanderbilt University Medical Center (VUMC) approved all study procedures, and the study was conducted in accordance with the US Federal Policy for the Protection of Human Subjects. Between October 2015 and November 2016, men with metastatic prostate cancer were recruited from urology and medical oncology clinics at VUMC and from the ZERO website. ZERO is a nonprofit organization that specifically supports men with prostate cancer by providing patient and caregiver education and advocacy activities. Eligible men had received a diagnosis of metastatic prostate cancer and agreed to complete a questionnaire in English about treatment‐related decision‐making and supportive management. After providing informed consent, participants completed surveys via REDCap describing their sociodemographic and clinical characteristics and their expectations for benefit from their current prostate cancer treatment, and optimism, among other items. The data that support the findings of this study are available from the corresponding author upon reasonable request.

## 3 MEASURES

### 3.1 Sociodemographic and clinical characteristics

Patients self‐reported their sociodemographic characteristics including age, race/ethnicity, marital status, employment status, health insurance status, and living situation. Patients also reported their self‐perceived overall health (“How would you describe your overall health right now?”) with the following response options: *excellent*, *good*, *fair*, and *poor*. Finally, patients self‐reported their most recent prostate‐specific antigen test result.

### 3.2 Expectations for benefit from treatment

Three survey items were adapted from items previously validated in incurable cancer populations and initially used in the Los Angeles Women's Health Study.^13^ Each item anchored to the patient's current treatment regimen (“After talking with your doctor about your current treatment, how likely do you think it is that it will*…*”) and assessed patient‐perceived likelihood of cure (“…cure your cancer?”), symptom relief (“…help with problems you are having because of your cancer?”), and life extension (“…help you live longer?”). Response options were *very likely*, *somewhat likely*, *a little likely*, *not at all likely*, and *don't know*.

### 3.3 Optimism

On the six‐item Life Orientation Test‐Revised, participants indicated their agreement with statements such as “in uncertain times, I usually expect the best” on a Likert scale from *strongly agree* (0) to *strongly disagree* (4). After appropriate reverse scoring, items were summed so higher scores represent greater optimism. Cronbach's alpha was acceptable (0.83).

### 3.4 Statistical analyses

Descriptive statistics (ie, means, standard deviations, and frequencies) were used to characterize the sample's sociodemographic characteristics, and differences by recruitment site were explored with independent samples t‐tests and chi‐square analyses. Frequencies were used to describe participants' expectations for benefits from treatment for metastatic prostate cancer. Fisher's exact tests were used to assess the associations of patient sociodemographic characteristics with expectations for treatment benefits. However, it was not possible to assess the associations of patient characteristics with expectations for prolonged life expectancy, because only one participant reported that this benefit was not at all likely. For each remaining outcome of interest (ie, expectations for cancer cure and symptom relief), the following patient characteristics were assessed: age (<70 years old vs ≥ 70), race (non‐white vs white), marital status (married or partnered vs not married or partnered), employment status (working vs retired), living situation (alone vs with others), self‐reported health status (excellent or good vs fair or poor), and optimism (< 20 vs ≥ 20). Significance was determined by a two‐sided alpha of less than 0.05. Although age and optimism are continuous variables, they were dichotomized for the Fisher's exact tests at a cut‐point of less than 70 years old vs greater than or equal to 70 years old and at the median optimism value of less than 20 vs greater than or equal to 20.

## 4 RESULTS

### 4.1 Sample characteristics

In total, 100 men consented to participate in this study (VUMC n = 77, ZERO n = 23). See Table 1 for all participant characteristics and comparisons by recruitment site. Average age was 68.33 years old (SD = 8.70) and participants were predominantly white (88%), married or partnered (80%), and had health insurance (99%). The most common site of disease metastasis was bone (79%), and most men reported one site of metastasis (77%). The majority of participants self‐reported an excellent or good health status (68%). Men recruited from VUMC and ZERO were not statistically different with regard to any of the sociodemographic variables, with the exception that men recruited from ZERO were more likely to be retired (*χ* ^2^(1)=6.02, *P* = .01).

**Table 1:** Descriptive sample characteristics and differences by recruitment site

Variable	Total sample (N = 100)	Recruitment Site		*P*‐value
		VUMC (n = 77)	ZERO (n = 23)	
Age, years; M (SD)	68.33 (8.70)	68.23 (8.42)	68.71 (0.86)	.82
Race/ethnicity; n (%)				.55
White	88 (88.0)	69 (89.6)	19 (82.6)	
Black/African American	10 (10.0)	7 (9.1)	3 (13.0)	
Hispanic/Latino	2 (2.0)	1 (1.3)	1 (4.3)	
Marital status; n (%)				.42
Married or partnered	80 (80.0)	62 (80.5)	18 (81.8)	
Single	8 (8.0)	5 (6.5)	3 (13.6)	
Widowed	6 (6.0)	6 (7.8)	0 (0.0)	
Divorced	5 (5.0)	4 (5.2)	1 (4.5)	
Employment status; n (%)				.01
Working	35 (35.0)	32 (42.1)	3 (13.6)	
Retired	63 (63.0)	44 (57.9)	19 (86.4)	
Health insurance status; n (%)				.58
Insured	99 (99.0)	76 (98.7)	23 (100.0)	
Not insured	1 (1.0)	1 (1.3)	0 (0.0)	
Living situation; n (%)				.42
Alone	13 (13.0)	9 (11.7)	4 (18.2)	
With spouse/partner or others	86 (86.0)	68 (88.3)	18 (81.8)	
Self‐reported health; n (%)				.06
Excellent or good	68 (68.0)	57 (74.0)	11 (52.4)	
Fair or poor	30 (30.0)	20 (26.0)	10 (47.6)	
Optimism; M (SD), possible range 0‐24	19.56 (4.31)	19.99 (4.19)	18.17 (4.49)	.08
Most recent PSA, ng/mL; M (SD)	12.33 (23.66)	10.99 (23.25)	16.41 (24.95)	.36
Site of metastasis; n (%)				
Bone	79 (79.0)	63 (81.8)	16 (69.6)	.21
Lymph node	27 (27.0)	20 (26.0)	7 (30.4)	.67
Liver	2 (2.0)	1 (1.3)	1 (4.3)	.36
Lung	3 (3.0)	2 (2.6)	1 (4.3)	.67
Other	3 (3.0)	1 (1.3)	2 (8.7)	.07
Don't know	7 (7.0)	6 (7.8)	1 (4.3)	.57
Total number of metastases; n (%)				.49
1	77 (77.0)	58 (81.7)	19 (86.4)	
2	11 (11.0)	10 (14.1)	1 (4.5)	
3	5 (5.0)	3 (4.2)	2 (9.1)	
Don't know	7 (7.0)	6 (7.8)	1 (4.3)	

Note

Variables with missing data are age (n = 96), marital status (n = 99), employment status (n = 98), living situation (n = 99), self‐reported health (n = 98), most recent PSA (n = 97), and optimism (n = 98).

Abbreviations: M, mean; PSA, prostate‐specific antigen; SD, standard deviation; VUMC, Vanderbilt University Medical Center; ZERO, website for men with prostate cancer.

### 4.2 Patient expectations of treatment benefits

#### 4.2.1 Cancer cure

**Figure 1:**
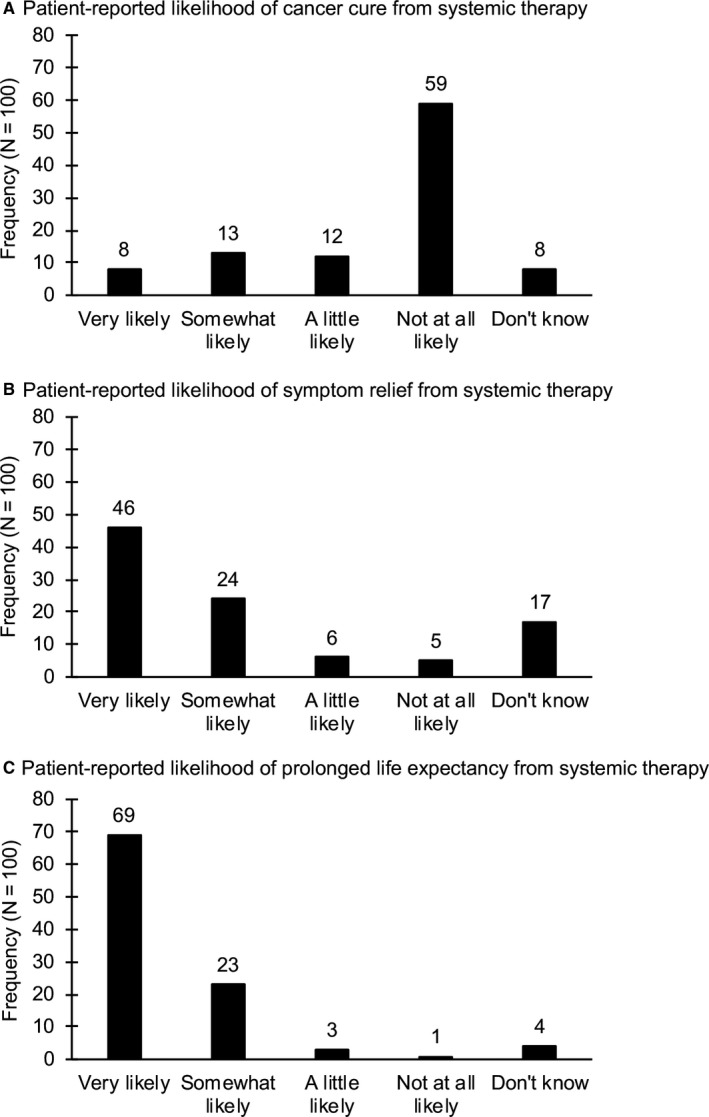
Responses to questions about the likelihood of benefit from systemic therapy for metastatic prostate cancer related to (A) cancer cure, (B) symptom relief, and (C) prolonged life expectancy

Overall, 41% of participants reported beliefs inconsistent with the understanding that their cancer was incurable, and this did not differ by recruitment site (*χ* ^2^(2) = 0.10, *P* = .95). A total of 33 men (33%) reported that their cancer treatment was at least a little likely to cure their metastatic prostate cancer (8% very likely, 13% somewhat likely, 12% a little likely) and 8 men (8%) did not know. By contrast, 59% of participants reported that their cancer treatment was not at all likely to cure their metastatic prostate cancer (Figure 1A).

#### 4.2.2 Symptom relief

Most participants (76%) reported that their cancer treatment was at least a little likely to provide symptom relief (46% very likely, 24% somewhat likely, 6% a little likely), whereas only 5% of participants reported that their cancer treatment was not at all likely to provide symptom relief, and 17% of participants did not know (Figure 1B). Expectations for symptom relief did not differ by recruitment site (*χ* ^2^(2) = 1.53, *P* = .47).

#### 4.2.3 Prolonged life expectancy

Almost all participants (95%) reported that their cancer treatment was at least a little likely to extend their life (69% very likely, 23% somewhat likely, 3% a little likely), with only 5% of participants collectively reporting that this benefit was not at all likely (1%) or unknown (4%; Figure 1C). Expectations for prolonging life expectancy did not differ by recruitment site (*χ* ^2^(2)=3.40, *P* = .18).

### 4.3 Patient characteristics associated with expectations of treatment benefits

See Table 2 for all associations between patient sociodemographic characteristics and patient beliefs regarding the likelihood of benefit from systemic therapy related to cancer cure (Table 2A) and symptom relief (Table 2B). The relationships between patient characteristics and the perceived likelihood of prolonged life expectancy could not be explored, because only one participant reported that this benefit was not at all likely.

**Table 2:** Fisher's exact tests assessing associations of patient characteristics with the expectations that benefits from systemic therapies for metastatic prostate cancer were at least a little likely vs not at all likely

Patient characteristic	(A) Cancer cure			(B) Symptom relief		
	At least a little likely	Not at all likely	*P*‐value	At least a little likely	Not at all likely	*P*‐value
	n (%)	n (%)		n (%)	n (%)	
Age			.82			.01
<70 y old	19 (61%)	33 (58%)		46 (62%)	0 (0%)	
≥70 y old	12 (39%)	24 (42%)		28 (38%)	5 (100%)	
Race			.01			.99
White	25 (76%)	56 (95%)		66 (87%)	5 (100%)	
Non‐white	8 (24%)	3 (5%)		10 (13%)	0 (0%)	
Marital status			.57			.06
Married or partnered	25 (78%)	50 (85%)		61 (81%)	2 (40%)	
Not married or partnered	7 (22%)	9 (15%)		14 (19%)	3 (60%)	
Employment status			.99			.15
Working	11 (35%)	20 (34%)		28 (38%)	0 (0%)	
Retired	20 (65%)	39 (66%)		46 (62%)	5 (100%)	
Living situation			.99			.53
With others	28 (88%)	51 (86%)		65 (87%)	4 (80%)	
Alone	4 (12%)	8 (14%)		10 (13%)	1 (20%)	
Health status			<.01			.99
Excellent or good	28 (90%)	34 (58%)		52 (70%)	4 (80%)	
Fair or poor	3 (10%)	25 (42%)		22 (30%)	1 (20%)	
Optimism score			<.01			.64
<20	7 (21%)	30 (53%)		31 (41%)	1 (25%)	
≥20	26 (79%)	27 (47%)		44 (59%)	3 (75%)	

Note

Age was dichotomized at a cut‐point of 70 y old. Optimism was dichotomized at the median value of 20.

#### 4.3.1 Cancer cure

Among men who reported that systemic therapy was at least a little likely to cure their cancer vs not at all likely to cure their cancer, there was a greater proportion of men who self‐identified as being of non‐white race (24% vs 5%, respectively; *P* = .01), who self‐reported excellent or good health (90% vs 58%, respectively; *P* < .01), and who had greater optimism (78% vs 47%, respectively, *P* < .01). Other factors examined (ie, age, marital status, employment status, and living situation) were not associated with expecting cure from systemic therapy.

#### 4.3.2 Symptom relief

Among men who reported that systemic therapy was at least a little likely to provide symptom relief vs not at all likely to provide symptom relief, there was a greater proportion of men who were less than 70 years old (62% vs 0%, respectively; *P* = .01). At the trend level, there was a greater proportion of men who were married or partnered who believed that systemic therapy was at least a little likely to provide symptom relief vs not at all likely (81% vs 40%, respectively), although this relationship did not reach statistical significance (*P* = .06). Other factors examined (ie, age, race, living situation, self‐reported health, and optimism) were not associated with greater likelihood of expecting symptom relief from systemic therapy. Of note, these associations should be interpreted cautiously due to the small number of men who reported that systemic therapy was not at all likely to provide symptom relief.

## 5 DISCUSSION

In a sample of men with metastatic prostate cancer, 33% of men reported inaccurate expectations that systemic therapies could possibly provide a cure for their incurable cancer. Men who held this inaccurate belief included a greater proportion of men who self‐identified as non‐white, self‐reported excellent or good health status, and had greater optimism in relation to men who held the accurate belief that treatment as not at all likely to cure their cancer. However, the majority of men reported accurate expectations that systemic therapies could provide symptom relief (76%) and prolong their life expectancy (95%), and a greater proportion of men younger than age 70 endorsed accurate expectations for symptom relief (vs inaccurate expectations). However, the association between younger age and expectations for symptom relief should be interpreted with caution, as only a small proportion of our sample reported that symptom relief was not at all likely as a result of systemic therapy.

Our findings are consistent with other studies showing that large proportions of patients with metastatic cancers hold beliefs inconsistent with the incurable nature of the disease.^6^, ^7^, ^8^ Having an accurate and realistic understanding of the potential risks and benefits for treatment is critical so that patients can make informed decisions about their care, and in the context of incurable disease, accurate expectations for cancer cure may be of highest priority. Indeed, past works show that patients are willing to accept toxic treatments for very small chances of cure, but may not accept the same treatments for lesser benefit (ie, life prolonging but not curative).^14^, ^15^ Moreover, patients with unrealistic beliefs about prognosis are more likely to pursue aggressive treatments.^16^ Thus, inaccurate beliefs about the possibility of cure could prohibit patients from making well‐informed decisions about their care and lead them to accept toxic treatments at the cost of quality of life. With these data, we showed that particular subgroups of men with metastatic prostate cancer may be most vulnerable to this possibility, namely non‐white men, men with better self‐perceived health, and men with greater optimism. Notably, the majority of participants described their overall health as excellent or good, despite having late stage cancer. Providers should consider these factors when interacting with this population, as these subgroups of men may need additional support to better understand the potential benefits and limitations of treatments for incurable disease.

Evidence suggests that discrepancies between metastatic cancer patients' and providers' expectations for cure could be related to providers' and patients' hesitancy to engage in discussions of prognosis as well as barriers to the patients' ability to understand or accept prognostic information.^17^ Discussing prognosis is a sensitive and delicate task that is affected by patients' preferences for such discussions as well as providers' communication styles. Patient preferences for prognostic information vary by individual and can be affected by factors such as cultural beliefs,^18^, ^19^ available coping strategies, and familial support.^20^ Patient‐oriented approaches to prognostic discussions that consider the individual patient's needs may be best suited for this task. Furthermore, it is critical that prognostic disclosure occur with the provision of hope,^21^ although in the face of incurable disease hope does not necessarily mean hope for cure. Rather, hope may be framed around more realistic treatment benefits that our data show patients are already largely aware of, including hope for symptom relief and hope for prolonged life expectancy.

To our knowledge, this is the first study to describe the expectations for treatment benefit among men with incurable metastatic prostate cancer. However, our findings should be interpreted in the context of the study's limitations. This sample of men with metastatic prostate cancer was largely white, partnered, and insured. In addition, optimism was high, with the average optimism score greater than 19 out of a total possible 24. Thus, it is possible that selection and other confounding biases affected the associations we observed. Finally, we did not have access to other sociodemographic (eg, highest level of education, total household income) and clinical information (eg, time since initial prostate cancer diagnosis) that could affect the associations described here, and our small sample size and limited statistical power may have affected our ability to detect differences in treatment expectations by patients' sociodemographic characteristics. Therefore, these findings are descriptive and largely hypothesis generating.

Nevertheless, these findings provide a substantial contribution to the literature describing cancer patients' expectations for treatment benefit in the context of treatment for incurable disease that often includes multiple lines of treatment. We showed that patients with metastatic prostate cancer endorse both accurate and inaccurate expectations for benefit from systemic therapies. Most concerning, one third of men endorsed inaccurate expectations about the curative potential of treatment, and this inaccurate belief was more common among non‐white men, men with better self‐perceived health, and men with greater optimism. Future research should focus on methods to align patient expectations with historically expected outcomes. Such alignment of expectations may lead to more informed shared decision‐making among patients and aid patients in making treatment decisions more consistent with their overall goals of care and personal preferences.

## ACKNOWLEDGMENTS

This work was supported by AHRQ training grant 1K12HS022990‐01 and NIH/NCATS CTSA award UL1TR000445. The author LBO was funded by NIH/NCI training grant T32CA193193. The authors gratefully acknowledge the individuals who participated in this study.
